# Trial, Error, and Insight: Using the Pilot Study of the HOPE Program to Inform Next Steps for Digital Single‐Session Research for Eating Disorders

**DOI:** 10.1002/eat.24487

**Published:** 2025-06-17

**Authors:** Bridianne O'Dea, Madelaine K de Valle

**Affiliations:** ^1^ Flinders University, Institute for Mental Health and Wellbeing, College of Education, Psychology and Social Work Adelaide South Australia Australia; ^2^ Blackbird Initiative Flinders University Adelaide South Australia Australia

**Keywords:** digital, eating disorders, mental health services, students

## Abstract

Digital single‐session interventions (D‐SSIs) are emerging as promising tools for bridging treatment gaps in youth mental health. Negi and Forbush's recent pilot study introduces “Help for Overcoming Problem Eating” (HOPE), a D‐SSI targeting binge‐spectrum eating disorders in university students. Their findings suggest preliminary acceptability, feasibility, and symptom improvements, contributing to the growing evidence‐base for D‐SSIs in disordered eating. This commentary outlines five key challenges that must be addressed to translate pilot findings into real‐world impact. First, the uptake of D‐SSIs is often low, likely due to help‐seeking barriers and septicism towards this approach. Peer‐led, promotional campaigns may boost engagement of D‐SSIs in young people. Second, despite their brevity, D‐SSI completion is not assured. Strategies such as automated reminders, peer or AI‐guided support, and youth co‐design may improve adherence. Third, economic evaluations are rare but critical for determining whether D‐SSIs offer cost‐effective support within resource‐limited student services. Fourth, trials should examine mechanisms and durability of change, with complementary D‐SSIs offering promising directions. Finally, meaningful integration into care pathways is critical. D‐SSIs may serve as stand‐alone supports, early engagement tools, or components of stepped‐care models. Lack of response could signal the need for intensive care, while successful use may increase openness to further help‐seeking. Addressing these challenges with informative, implementation‐ready trials will be key to realizing the full potential of D‐SSIs in addressing eating disorders in university students and advancing youth mental health care more broadly.

## 1 Introduction

Digital single‐session interventions (D‐SSIs) have rapidly gained traction as a promising approach to improving mental health outcomes and addressing treatment gaps across several mental health disorders, populations, and settings. In their recent study, Negi and Forbush introduce “Help for Overcoming Problem Eating” (HOPE), a D‐SSI designed specifically for university students with binge‐spectrum eating disorders (EDs) (Negi and Forbush 2025). Their pilot demonstrated aspects of feasibility, acceptability, and preliminary benefits for some ED symptoms, including binge eating, restricting, and cognitive restraint. The effect sizes observed suggest that HOPE may provide meaningful symptom relief, at least in the short term. These findings contribute to the growing literature on the efficacy of D‐SSIs for reducing disordered eating behaviors in young people. While the pilot results are promising, a randomized controlled trial (RCT) or equivalent is the essential next step for establishing the viability of this approach for addressing the rising rates of disordered eating in young people. Informed by prior work, we propose five key challenges that must be addressed in future trial planning to accelerate the translation of D‐SSIs into real‐world impact.

## 2 Challenge 1: Initiating Uptake of D‐SSIs

A core advantage of D‐SSIs is the greater potential for broad reach due to increased Internet access, the minimal time commitment, and reduced therapeutic intensity of these programs. Improving treatment accessibility is a particularly salient consideration for people with eating disorders, who generally demonstrate low levels of help‐seeking. The low time demands of D‐SSIs may appeal to university students who often work long hours as time and effort are key factors influencing working adults' intentions to use digital mental health interventions (DMHIs). However, initiating uptake of DMHIs among young people remains a key challenge in research trials and real‐world settings (Boucher and Raiker 2024). Despite the brevity of the HOPE program, Negi and Forbush (2025) found that only 42.6% (*n =* 81/190) of eligible university students chose to access it. Understanding why this was the case is critical for refining outreach strategies and improving university students' engagement with D‐SSIs in trials and beyond.

To date, trials of D‐SSIs have not provided conclusive insights into how intrinsic and extrinsic motivational factors intersect with mental health literacy, treatment beliefs and expectations, and other barriers and facilitators, to shape young people's help‐seeking for EDs. While university students may appreciate the privacy and convenience afforded by D‐SSIs, stigma, denial, and shame may still deter them from using digital supports for binge eating (Daugelat et al. 2023). To increase help‐seeking and the uptake of D‐SSIs for EDs among university students, there may be value in testing adjunctive on‐campus social marketing campaigns that increase students' awareness and understanding of EDs. Such campaigns could adopt weight‐neutral, health‐enhancing self‐care messages and emphasize the importance of selecting evidence‐based treatments for weight‐related health concerns. Given the role of motivation in help‐seeking for EDs (Daugelat et al. 2023), strategic marketing of D‐SSIs could be strengthened by adopting a transtheoretical “stages of change” model that incorporates anti‐stigma content, lived experience narratives, and motivational interview techniques, to engage students in the contemplation stage who may prefer low‐intensity treatment as a first step. Peer‐led campaigns may also enhance D‐SSI uptake among university students, as positive social support can facilitate treatment engagement for EDs and aligns with the socially interactive nature of university (Daugelat et al. 2023). Future research should explore how social networks within universities can be leveraged to promote help‐seeking for EDs.

Limited openness to digital therapies and misbeliefs about effectiveness may further inhibit the uptake of D‐SSIs among university students, particularly in on‐campus clinical settings where in‐person care may be expected or prioritized. In their systematic review, Zhu and colleagues (Zhu et al. 2025) identified perceived quality and effectiveness as key facilitators (and barriers) to young people's use of DMHIs. Targeted strategies to increase mental health literacy and reduce skepticism may enhance university students' uptake of D‐SSIs. Offering students' choice may further enhance uptake. Future trials should aim to empirically validate these barriers and facilitators using data‐driven methods, including assessment of students' attitudes towards digital therapies and experimental designs to evaluate strategies for promoting uptake. Such findings will be critical to informing targeted approaches for integrating D‐SSIs into university mental health services.

Another potential strategy to increase the uptake of D‐SSIs for EDs among university students is to offer programs that target transdiagnostic processes rather than disorder‐specific symptoms. This approach is supported by trials of other D‐SSIs, which have reduced restrictive eating behaviors in young people without program content directly targeting EDs (Schleider et al. 2022). The academic pressures of university life may make transdiagnostic processes such as perfectionism, self‐criticism, and low self‐esteem particularly salient to young adults. However, Wade and colleagues identified 38 distinct transdiagnostic processes relevant to EDs in young people, which may represent novel targets for future D‐SSIs (Wade et al. 2025). Marketing D‐SSIs in terms of these broader psychological processes—rather than explicitly referencing EDs—may help to overcome some of the barriers to help‐seeking posed by stigma, denial, and shame.

## 3 Challenge 2: Ensuring Completions of D‐SSIs

A major strength of D‐SSIs compared to multi‐sessional digital therapies is the greater potential for completion due to lower time demands. In Negi and Forbush's pilot, 92.6% completed the HOPE program (*n* = 75/81). Completer feedback indicated that HOPE was engaging, easy to use, and beneficial. However, although HOPE was designed as a single‐session program, many students completed it over several days, sometimes up to a week, rather than in a single sitting. This suggests that reduced time commitment alone may not be sufficient to drive completion of DMHIs. It also raises important questions about how we distinguish truly single‐session DMHIs from brief, multi‐part programs completed over multiple sittings. Clarifying this distinction is critical for future intervention and trial designs, particularly given real‐world completion rates for D‐SSIs remain low (Cohen and Schleider 2022). The effect size reported by Negi and Forbush (*ƞ* ^*2*^ = 0.01, see Table 1 in Negi and Forbush 2025) in article may indicate that completions of HOPE were somewhat affected by ED‐related impairment, but larger samples are needed to detect this. Future trials should investigate the role of functional impairment and assess the need for individual‐level support. Low‐cost prompts such as automated app notifications or SMS reminders, or manual outreach by peer workers, care navigators, or clinical staff, could help increase completion. Generative artificial intelligence (AI) coaches also warrant consideration. A more contentious strategy is to explore financial reimbursement as an incentive for D‐SSI completion. While previous research suggests this may increase uptake (Cohen and Schleider 2022), offering compensation for brief interventions without rigorous screening can result in fraudulent enrolment, necessitating strong safeguards (Davies et al. 2024). Finally, co‐designing D‐SSIs and promotional materials with young people with lived experience may help to maximize the appeal to university students.

## 4 Challenge 3: The Need for Adequately Powered Economic Evaluations of D‐SSIs

By designing HOPE as a digital, self‐guided SSI, the authors have attempted to provide a low‐cost intervention. Low‐cost interventions can be attractive to university clinical services, where demand is high, needs are diverse, but treatment resources are low. The viability of HOPE as a treatment offering will be significantly strengthened by the inclusion of an economic evaluation in subsequent RCTs, as the cost‐effectiveness of digital interventions for mental disorders is likely but not guaranteed. To date, there have been very few economic evaluations of D‐SSIs in young people. Future trials of HOPE may be strengthened by including a supplementary, adequately powered economic evaluation to support the economic benefits and investment in this approach.

## 5 Challenge 4: Examining Mechanisms and Durability of Effects of D‐SSIs in Diverse Populations

Pilot trials are key to identifying intervention and protocol improvements. As the number of D‐SSIs continues to grow, there is an increasing need for effective methods to assess and map the fidelity of intervention design and content to best‐practice principles, enabling ongoing product improvement. The four BEST principles used by Negi and Forbush are widely recognized as a clear, evidence‐informed framework for developing D‐SSIs and underpin several efficacious interventions. However, retrospectively identifying which components of D‐SSIs require refinement remains a challenge.

In the pilot, the HOPE program appeared to have limited impact on subjective binge eating and meal skipping. Many participants struggled to implement regular eating due to persistent body image concerns. This suggests that the program content may need to be expanded to address these factors. However, expanding the program's scope—and the time and effort required for completion—may not improve its effectiveness. Instead of expanding the content of HOPE, an alternative approach could involve offering individuals a suite of D‐SSIs, each targeting different barriers to change, allowing individuals to select those most relevant to their needs. This aligns with Negi and Forbush's suggestion of offering HOPE alongside other complementary interventions. Advances in machine learning and AI may also be used to personalize suggestions for next steps after D‐SSI completion, based on the outcomes or unmet needs that individuals report. Alternatively, exploring opportunities within the broader university context may lead to novel complementary interventions that enhance the effectiveness of D‐SSIs for mental health. Future research could explore how delivering complementary SSIs within broader university settings may prime or amplify the effectiveness of those offered through university mental health services.

While the HOPE pilot demonstrated significant symptom reductions, it remains unclear whether these changes resulted from the intervention itself or from natural symptom fluctuation. Future trials should aim to examine just how durable the effects of D‐SSIs are for ED‐related mental health outcomes in university students. Given the chronic nature of EDs, it is essential to understand whether a single session is sufficient or if booster sessions are necessary to maintain gains. Furthermore, the characteristics of participants in many digital mental health trials do not reflect the full spectrum of individuals affected by mental illness. Developing targeted recruitment strategies to reach more diverse populations who are experiencing binge EDs, including young adult males and students from culturally and linguistically diverse backgrounds, will help to reduce biases in our rapidly growing evidence base of D‐SSIs for young people.

## 6 Challenge 5: Finding the Fit for D‐SSIs in Service Models

The HOPE study contributes valuable insights into the feasibility of digital SSIs for EDs, but a key question remains—how should SSIs be integrated into service models? A challenge for disseminating D‐SSIs is appropriately positioning these interventions into the help‐seeking journey for young people with EDs. In many settings, digital interventions have failed to be integrated into care pathways in ways that are respected by consumers and honored by clinicians. Multi‐disciplinary teams involving service commissioners, service deliverers, and consumers are critical for tackling adoption. Health service design methodologies, including service blueprinting and co‐design with lived experience experts and service delivery experts, may help to identify the optimal positioning of D‐SSIs in services. Implementation trials can be helpful for testing out service delivery models but cannot eradicate all barriers to service‐level adoption and can be difficult to carry out due to administrative and bureaucratic barriers. Guided by this, future trials should aim to examine whether D‐SSIs like HOPE are effective as a standalone treatment or whether D‐SSIs are better integrated into multi‐tiered service models, such as stepped‐care approaches, where they can serve as early intervention, a gateway to more intensive treatments, or as a ‘step‐down’ or relapse prevention approach. In the context of EDs, it may be valuable to investigate whether low uptake or non‐response to D‐SSIs serves as a reliable indicator of the need for more intensive treatment. Conversely, D‐SSIs may also facilitate treatment engagement by increasing openness to care and providing an accessible first step. These possibilities highlight the potential of D‐SSIs to enhance youth mental health treatment pathways, particularly given the chronic nature of many mental illnesses and the need for varying levels of intervention intensity over time.

## 7 Concluding Remarks

The pilot study of the HOPE program contributes to the growing literature on D‐SSIs in the field of ED treatment, indicating that these brief interventions may be acceptable and effective for addressing binge eating in university populations. As DMHIs continue to evolve, studies like this pave the way for innovative, accessible solutions to address the growing burden of EDs among young adults. To accelerate progress in the field, it is crucial to apply insights from this pilot and learnings from other digital mental health trials, carefully addressing the challenges outlined above. The field of D‐SSIs would benefit from continued discussions and development of optimal trial designs for brief interventions that incorporate logic modeling and appropriate measures that can appropriately capture short‐ and long‐term changes. By doing so, future trials can generate meaningful evidence to advance the development and implementation of D‐SSIs for EDs and youth mental health more broadly.

## Author Contributions

**Bridianne O'Dea:** conceptualization, writing – original draft, writing – review and editing. **Madelaine K de Valle:** conceptualization, writing – original draft, writing – review and editing.

## Conflicts of Interest

The authors declare no conflicts of interest.

## Data Availability

Data sharing not applicable to this article as no datasets were generated or analysed during the current study.
